# Anatomically-specific intratubular and interstitial biominerals in the human renal medullo-papillary complex

**DOI:** 10.1371/journal.pone.0187103

**Published:** 2017-11-16

**Authors:** Ling Chen, Ryan S. Hsi, Feifei Yang, Benjamin A. Sherer, Marshall L. Stoller, Sunita P. Ho

**Affiliations:** 1 Division of Biomaterials and Bioengineering, Department of Preventive and Restorative Dental Sciences, School of Dentistry, University of California San Francisco, San Francisco, California, United States of America; 2 Department of Urologic Surgery, Vanderbilt University Medical Center, Nashville, Tennessee, United States of America; 3 Department of Urology, School of Medicine, University of California San Francisco, San Francisco, California, United States of America; Universita degli Studi di Bari Aldo Moro, ITALY

## Abstract

Limited information exists on the anatomically-specific early stage events leading to clinically detectable mineral aggregates in the renal papilla. In this study, quantitative multiscale correlative maps of structural, elemental and biochemical properties of whole medullo-papillary complexes from human kidneys were developed. Correlative maps of properties specific to the uriniferous and vascular tubules using high-resolution X-ray computed tomography, scanning and transmission electron microscopy, energy dispersive X-ray spectroscopy, and immunolocalization of noncollagenous proteins (NCPs) along with their association with anatomy specific biominerals were obtained. Results illustrated that intratubular spherical aggregates primarily form at the proximal regions distant from the papillary tip while interstitial spherical and fibrillar aggregates are distally located near the papillary tip. Biominerals at the papillary tip were closely localized with 10 to 50 μm diameter vasa recta immunolocalized for CD31 inside the medullo-papillary complex. Abundant NCPs known to regulate bone mineralization were localized within nanoparticles, forming early pathologic mineralized regions of the complex. Based on the physical association between vascular and urothelial tubules, results from light and electron microscopy techniques suggested that these NCPs could be delivered from vasculature to prompt calcification of the interstitial regions or they might be synthesized from local vascular smooth muscle cells after transdifferentiation into osteoblast-like phenotypes. In addition, results provided insights into the plausible temporal events that link the anatomically specific intratubular mineral aggregates with the interstitial biomineralization processes within the functional unit of the kidney.

## Introduction

Pathological mineral formations occur in various organ systems within the human body. Within the kidney, these are most commonly identified as a urinary tract stone. Little is known about the early stage biominerals that lead to clinically detectable stones, despite their increasing prevalence [1] and associated global health burden [2]. Mapping of physicochemical properties within tissues of the kidney could provide insights into the pathologic biomineralization processes and direct newer treatments to help prevent urinary tract stone formations. The novel aspects of this study will include multiscale evaluation of intact whole renal medullo-papillary complexes using high-resolution microscopy to localize minerals with precise anatomical specificity. Additionally, results will provide insights into plausible temporal events leading to stone formation.

The medullo-papillary complex is one of the 8–12, paraboloid-shaped functional units within a kidney. At the tip of the complex is the papilla, where the end-product, urine, drains into the urinary collecting system. Fundamentally, the renal medullo-papillary complex consists of both uriniferous and vascular tubules of various lengths and diameters where exchange of ions and water occurs along the length and across the tubules. Therefore, from a bioengineering perspective, the medullo-papillary complexes in a kidney can be thought of as biofilters in which solutes are separated from the solvents.

Investigations on the etiology of calcium oxalate-based kidney stones have focused on their attachment to biominerals known as the Randall’s plaque (RP) and are commonly observed (endoscopically and grossly) at the tip of the complex [3]. Stone formers have an increased area of RP coverage per papilla [4] and stones are often attached to the papilla [5], compared to that of non-stone formers. Therefore, endoscopically visible RP has been characterized as a clinically detectable precursor of calcium-based kidney stones.

RP has been characterized at multiple length-scales using various microscopy and spectroscopy techniques [6]. However, the processes that lead to RP formation are minimally understood. Furthermore, biomineralization processes deeper within the complex (not able to be observed endoscopically) beyond the papillary tip are unknown. The current thinking is that RP formation at the renal tip is an interstitial process that eventually leads to downstream events where a urinary stone formation progresses from RP within into the collecting system of the kidney [7–11]. Given this transition in environment, the question to ask is: what are the nanoscale precursors that lead to macroscale interstitial RP formations that are then identified endoscopically?

Several hypotheses involving cell-based and/or physical chemistry-based mineralization have been proposed to describe RP formations and the process of calcium-based stone formation and the association of the stone with RP [7, 8, 12]. Among these hypotheses, it has been suggested that the renal epithelial cells of uriniferous tubules [8, 12], similar to the differentiation of pericytes [13], smooth muscle cells [14], and endothelial cells [15] can also differentiate towards osteoblast-like cells prompting vascular mineralization. Several noncollagenous proteins (NCPs) such as osteopontin (OPN), osteocalcin (OC), and bone sialoprotein (BSP) identified in bone extracellular matrix have been identified in renal calculi and are synthesized by renal epithelial tubular cells [10, 12, 16]. Therefore, it has been proposed that these proteins indicate a plausible change in cell phenotype towards an osteoblastic lineage derived from renal membrane epithelial cells [12]. However, a recent study by Evan et al. suggests a different mechanism for the formation of RP and stones [17]. Other thoughts on the factors affecting the mineralization in renal papilla and subsequent stone formation can be found in several well-written review papers [18–20].

NCPs are known to sequester inorganic ions on organic matrices [21–26] and this mechanism is within the realm of physiologic biomineralization to accommodate dynamic mechanical loads on bone. The site-specific localization of these NCPs is appreciated when seen in the osteonal growth of bone centered around blood channels (Volkmann’s canals). This implies direct involvement of vasculature and its association in the formation of guided concentric mineralized layers: the laminates. Fundamentally, vascular elements are involved, but from a hierarchical length scale perspective, the NCPs and inorganic ions, and their interactions are guided through physical and chemical cues specifically within mechanoresponsive tissues. Given that several NCPs have been identified within RP [27, 28], the subsequent question to ask is, could the same NCPs associated with bone formation also be involved in biomineralization of the medullo-papillary complex more proximally within the renal parenchyma as a result of physical and chemical cues? Additionally, what is the association of the vasculature towards RP formation [29]. In this study, we hypothesize that the precursors in both intratubular (inside vasa recta or uriniferous tubules) and interstitial (matrix tissue surrounding vasa recta and uriniferous tubules) mineral formations consist of the same NCPs found in mineralization of soft (e.g. ligament) and hard (e.g. bone) tissues in the body. These NCPs can play a role in renal pathologic mineralization, and the structure of minerals and aggregates relevant to their organic templates are postulated to be anatomically-specific.

We aim to better understand how NCPs (OPN, OC, and BSP) affect the mineralization process in RP and subsequent stone formation. In this study, NCPs will be first localized within the anatomy of human renal medullo-papillary complexes. This is in contrast to prior studies that have focused exclusively at the tip of the renal papilla. In addition, we will examine the location of these NCPs relative to the location of vasculature and uriniferous tubules to provide insights into whether they are synthesized by renal epithelial cells or delivered through the vasa recta. Furthermore, it is equally critical to gain insights into how the location of the NCPs in the intratubular and interstitial regions affects the corresponding cascades of progressive, locally occurring biomineralization processes and subsequently the functional form of the complex.

## Materials and methods

All participants provided both verbal and written consents to participate in this study. Institutional approval for both the study itself and the manner in which consent was obtained from Human Research Protection Program (HRPP) at the University of California, San Francisco, was recorded under institutional review board (IRB) approval number 14–14533. Participants discussed the details of the study at their clinical visit where questions were answered by research team members and their verbal and written consents to participate were obtained. All study consents were then scanned and recorded into our data warehouse and the paper copies were destroyed in a secure fashion in accordance with institutional standards. Papillary specimens (n = 14) were obtained from patients undergoing nephrectomy for the diagnosis of renal mass, presumed to be renal cell carcinoma. Patients who were thought to have transitional cell carcinoma were excluded from this study. During harvest of the papillae, an anatomically normal portion of the kidney, distant from the renal mass, was selected. In accordance with current dogma, renal tissue distant from renal cell carcinoma is generally thought to be normal unless subject to mass effect from the tumor. Typically, if a lesion is small enough and it is possible to reconstruct the kidney after mass excision, partial nephrectomy is preferred according to AUA guidelines [30]. This is based upon a multitude of published literature suggesting that not only will renal tissue that is preserved continue to function at a near normal level, but that oncological outcomes are excellent in terms of local recurrence [31, 32]. Unfortunately, in this patient cohort, radical nephrectomy was indicated based upon tumor anatomy (for instance the tumor was too close to the blood vessels to allow safe excision, or reconstruction was not possible) and salvage of the kidney was not possible [33]. Neo-adjuvant chemotherapy was not employed in any patient before nephrectomy in accordance with standard of care. Excised tissue distant to the tumor was examined at nephrectomy, had a grossly normal appearance, and was felt to be appropriate for inclusion.

In this study, immunohistochemistry, fluorescence, and electron microscopy techniques were chosen to study the anatomy-specific biominerals in human renal complex. Mapping of biominerals at multiple length scales was performed by using X-ray, light, and electron imaging modalities to enable contextual visualization of vascular and uriniferous tubules at the level of the complex, and localization of globular proteins in the tubules and surrounding matrices through 3, 3'-diaminobenzidine (DAB), and gold-particle tagged probes at the level of a tissue (interstitium). Anatomy-specific and multiscale mapping of mineralized regions and localization of NCPs was performed in three zones (I-III) of the complex (S1 Fig).

### Specimen preparation for X-ray micro computed tomography (micro-CT), scanning electron microscopy (SEM), and energy dispersive X-ray spectroscopy (EDS)

Of the 14, one of the specimens was stained with iodine. The renal complex was carefully isolated from a fresh nephrectomy specimen, fixed overnight in 10% neutral buffered formalin (NBF, Richard-Allan Scientific, Kalamazoo, MI), washed three times in 1× phosphate-buffered saline (PBS), and was scanned using micro-CT before staining with iodine. Following scanning, the same papilla was stained with 1.5% iodine (Alfa Aesar, Ward Hill, MA) for 2 hours. After washing with ethanol three times, the stained papilla was scanned using micro-CT (MicroXCT-200, Carl Zeiss Microscopy, Pleasanton, CA). Following scanning, the specimen was embedded in paraffin, and was sectioned and examined using a light microscope (Olympus BX51). Micro-CT was performed at 4X magnification, 1200 slices, providing a resolution of 5 μm/pixel. X-ray scanning of specimens before and after iodine staining and superposition of the respective tomograms provided location and anatomical association of mineralized regions in those that were stained with iodine. It is known that vasculature can be revealed by iodine staining or iodinated contrast agents filling [34]. Intratubular minerals were difficult to retain during histology processing. Therefore, the best approach is to use X-ray tomogram illustrating the relation between intratubular minerals and vasculature highlighted from Iodine staining. X-ray tomograms of minerals and iodine stained vasculature will help understand their spatial correlation.

Medullo-papillary complexes containing RP (RP+) were dehydrated using graded (50–100%) ethanol solutions. The specimen was scanned using a micro-CT followed by infiltration of LR-white resin (Electron Microscopy Sciences, Hatfield, PA). The infiltrated specimen was kept in a gelatin capsule (Electron Microscopy Sciences) and polymerized for 2 days at 60°C. 90 nm thick tissue sections were cut with an ultramicrotome (Reichert Ultracut E, Leica Microsystems, Inc., Buffalo Grove, IL). The ultrasectioned block surface was sputter coated with a thin layer of gold/palladium, and was used for structural analyses using a field emission scanning electron microscope (SEM, Sigma VP500 field emission SEM, Carl Zeiss Microscopy) at 5 keV. The ultrasections were collected on formvar/carbon-coated Ni grids (Electron Microscopy Sciences) for immunohistochemical localization of NCPs. An energy dispersive X-ray spectroscopy (EDS) detector (Bruker AXS, Madison, WI) was used for elemental analyses of histology sections from iodine stained papilla without RP (RP-) placed on ITO (indium tin oxide) coated glass coverslips. Using SEM and EDS analysis, the region rich in iodine can be correlated to the specific structures from morphological analysis.

### Polarized and fluorescent microscopy techniques, and immunohistochemistry on tissue sections of the medullo-papillary complex for noncollagenous protein localization using light and transmission electron microscopy techniques

Following micro-CT, sections of papillary tissue showing RP were imaged using polarized and fluorescent microscopy (Olympus BX51) before and after 1 hour of 4% EDTA treatment. Rabbit polyclonal antibody against human OC (sc-30044), mouse monoclonal antibodies for BSP (sc-73630) and OPN (sc-21742) were purchased from Santa Cruz Biotechnology, Inc. (Dallas, TX). Mouse monoclonal antibody against human CD31 (ab9498) was acquired from Abcam, (Cambridge, MA). Rabbit polyclonal antibody against human BSP (LF-84) was received as a gift from Larry W. Fisher, (Craniofacial and Skeletal Diseases Branch, NIDCR/NIH). Goat polyclonal antibody against human OPN (AF-1344) was obtained from R&D System, Inc. (Minneapolis, MN).

The medullo-papillary complexes with (RP+) and without visible RP (RP-) (5 specimens) were processed using 15% and 30% sucrose solutions and then embedded in optimal cutting temperature (OCT) resin. 6–7 μm thick sections were cryo-sectioned and collected on glass slides. Immunohistochemistry (IHC) labeling of NCPs and endothelial cell adhesion protein CD31 was performed on sections decalcified overnight in 4% EDTA solution. The sections were incubated in antibody solution (1/200 dilution for all antibodies except that 1/500 was used for mouse monoclonal antibody against OPN). As for IHC staining of CD31, a heat epitope retrieval process in citrate buffer (pH = 6) at 80°C for 20 min was applied. The control test was performed without addition of primary antibody in parallel. Afterwards, the sections were stained using secondary antibody kits (Vectastain Elite ABC, Vector Laboratories, Inc., Burlingame, CA) and DAB substrate kit (ImmPACT DAB peroxidase substrate, Vector Laboratories, Inc.). After counter-staining using hematoxylin, the sections were dried and imaged using a light microscope (BX51, Olympus Scientific Solutions Americas Corp. Waltham, MA). The localization of NCPs was similar among all specimens and representative data are shown in this manuscript (see Results).

Immunolocalization of NCPs was performed through immunogold labeling of thin sections on nickel grids using polyclonal antibodies [35]. The ultrasections were decalcified with 4% EDTA solution for 20 min. Decalcified sections were washed twice using Milli-Q water, twice with PBS, and were treated with a blocking agent containing 2.5% bovine serum albumin (BSA, Sigma-Aldrich) in PBS for 10 min. Grids were incubated in primary antibody solution (1:20 dilution in PBS) at 4°C overnight. After washing three times with PBS, sections were treated again with 2.5% BSA for 10 min then incubated in a 10-nm-diameter protein G-gold nanoparticle solution (Electron Microscopy Sciences, Hatfield, PA) for 1 hr at room temperature. Following washing three times with PBS, grids were rinsed with Milli-Q water and air-dried overnight. No counterstaining was applied to the sections. The sections were examined using a transmission electron microscope (JEOL USA, Inc., Peabody, MA) operated at an accelerating voltage of 120 keV.

## Results

### Spatial distribution of minerals and their association with tubules of the renal medullo-papillary complex

**Fig 1:**
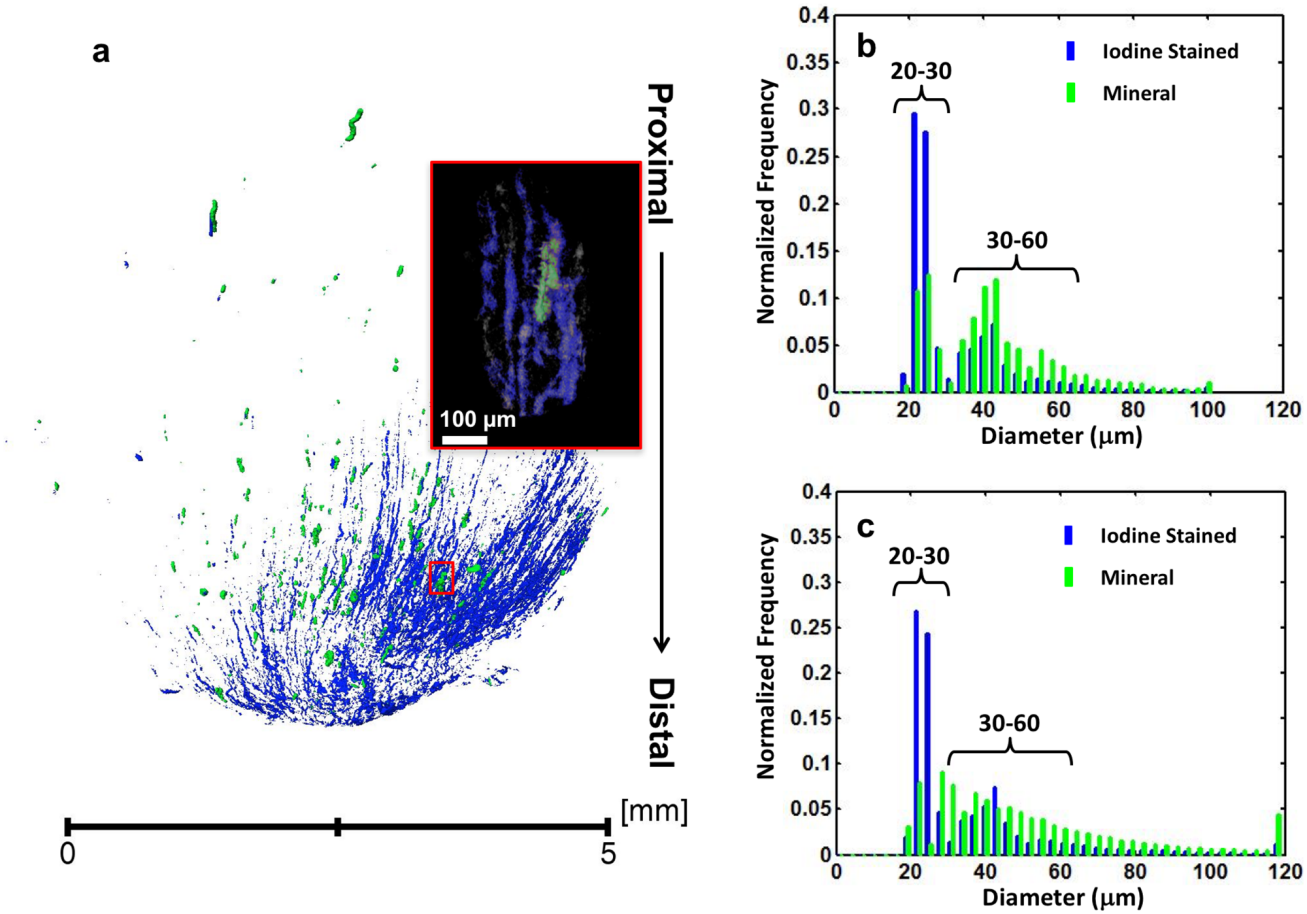
X-ray tomography of a human medullo-papillary complex with intratubular mineralization and without identifiable Randall’s plaque (RP-). (a) X-ray tomograms of unstained and iodine stained specimens were superimposed to illustrate the overlap between the mineralized tubules (green) and radiopaque iodine-stained tubules (blue). In the inset, a representative mineralized tubule (green) is co-localized with iodine stained tubules as visualized with a micro-CT scan at a higher magnification. The corresponding location of the inset is highlighted with red rectangle in the X-ray tomogram. The mineralized and iodine stained tubules were digitally segmented based on the mineral density difference and confirmed within X-ray scans performed on pre and post-iodine stained of the same specimen (b). In addition, (b) a histogram of the diameters of the mineralized (green) and iodine stained (blue) tubules of pre- and post-iodine stained specimens illustrated a bimodal distribution indicating two distinct diameters within the ranges of 20–30 μm and 30–60 μm (c).

Intratubular mineralized structures were consistently observed in the proximal, peripheral regions distant from the papillary tip in all specimens, irrespective of whether RP was identified at the papillary tip. The superposition of tomograms acquired before and after iodine-staining (Fig 1a) suggested that mineralized tubules spatially overlapped with iodine stained tubules for which the pixel resolution was about 5 microns. Data obtained from higher magnification clearly show the co-localization of mineralized and iodine stained tubules (inset of Fig 1a and supplemental video S2 File). As different renal tubules have distinct sizes, the tubular diameter can serve as an indicator of the tubular nature. For example, the diameter of uriniferous tubules is about ten times of a vasa recta. After analyzing the diameter of the mineralized and iodine stained tubules, their distributions were similar (Fig 1b). Two major peaks were observed between 20–60 μm indicating two distinct mineralized populations of tubules; ranges included 20–30 μm and 30–60 μm.

An additional five unstained RP- papillae were analyzed using the same approach. The combined data indicated that the majority of mineralized tubules within these proximal regions have a diameter between 20–60 μm (Fig 1c) and the two aforementioned ranges were also observed.

**Fig 2:**
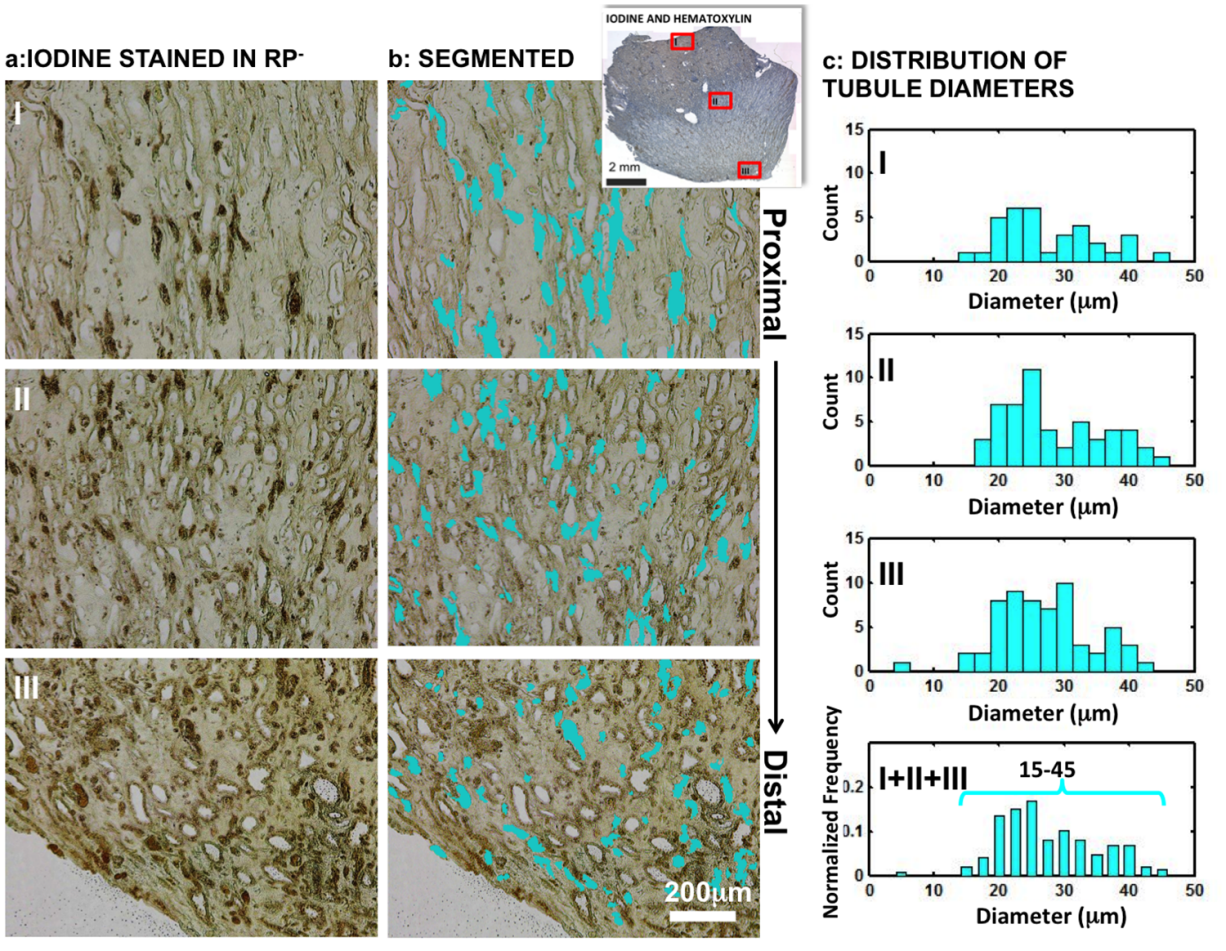
Light microscopy images of histology sections and segmented diameters obtained from iodine stained human papilla without identifiable Randall’s plaque (RP-). (a) The tubules stained with iodine were digitally segmented (b, cyan) and (c) the corresponding distributions of tubule diameters are plotted in the histograms. The red rectangles in the inset represent the three zones (I, II, and III) of interest and correspond to the most proximal region of the medullopapillary complex (I), the mid-region of the medullo-papillary complex (II), and papillary tip (III) respectively. The histograms of the iodine stained tubule diameters for each zone were plotted separately. The bottom right histogram shows an overall normalized distribution of stained tubular diameter, which was also plotted from the summary of all three zones (I+II+III). The histograms indicate the stained tubular diameters were mainly in the range between 20 and 40μm in different zones.

Tubules preferentially stained with iodine histologically examined (Fig 2a) and were digitally segmented (Fig 2b). The diameters of these tubules were also within a range of 20–40 μm (Fig 2c) in different distinct locations within the proximal to the distal part of the papilla (inset, I-III) (S1 Fig). In summary, based on histomorphometry of iodinated sections using light and X-ray tomograms, a significant overlap between the iodine stained and mineralized tubules was observed, indicating these tubules are the same. Tubules that did not retain iodine were of a larger diameter than those with iodine (I-III).

**Fig 3:**
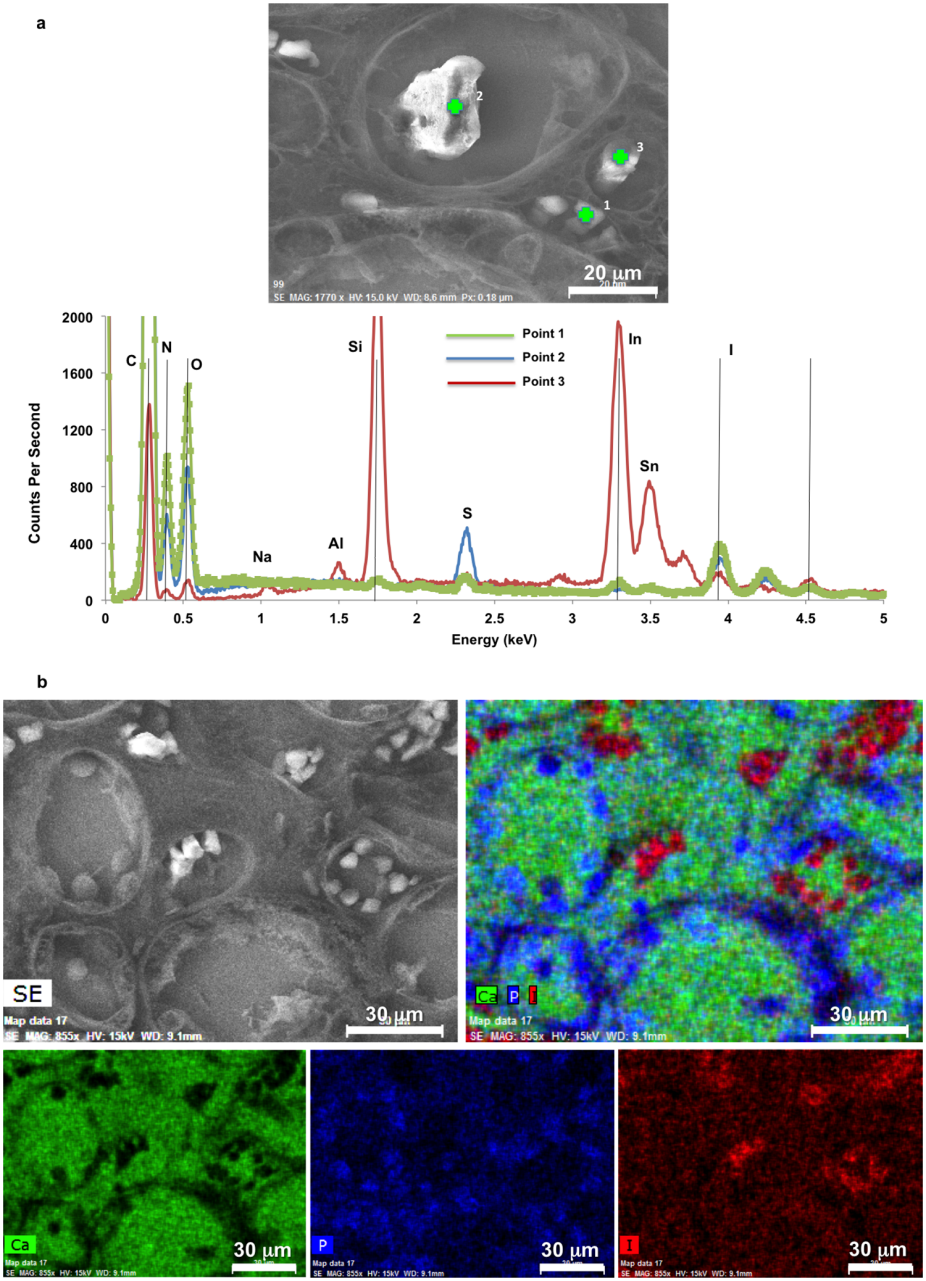
SEM and EDS analysis of a histology section obtained from the same iodine stained papilla helped further localize iodine in vasa recta (identified by erythrocytes inside the vasa recta, regions 1–3). (a) The corresponding EDS spectra of three regions suggested the erythrocyte-like structures were preferentially stained with iodine. Characteristic X-ray energy peaks of elements such as silicon (Si), tin (Sn), and indium (In) in EDS spectra were from the ITO (indium tin oxide) coated on the glass substrate. Other elements such as C, N, and S are associated with tissue and partially from residual paraffin on the histology section. (b) A SEM image taken from a different region with corresponding area maps for Ca (green), P (blue), and I (red) elements are shown. Both individual and correlated elemental maps of the same region were obtained. Erythrocytes showed higher iodine localization, indicating the iodine preferentially stains the erythrocyte. Other cellular structures associated with tubules showed higher phosphorus content. Calcium was distributed evenly both within and outside tubular structures.

**Fig 4:**
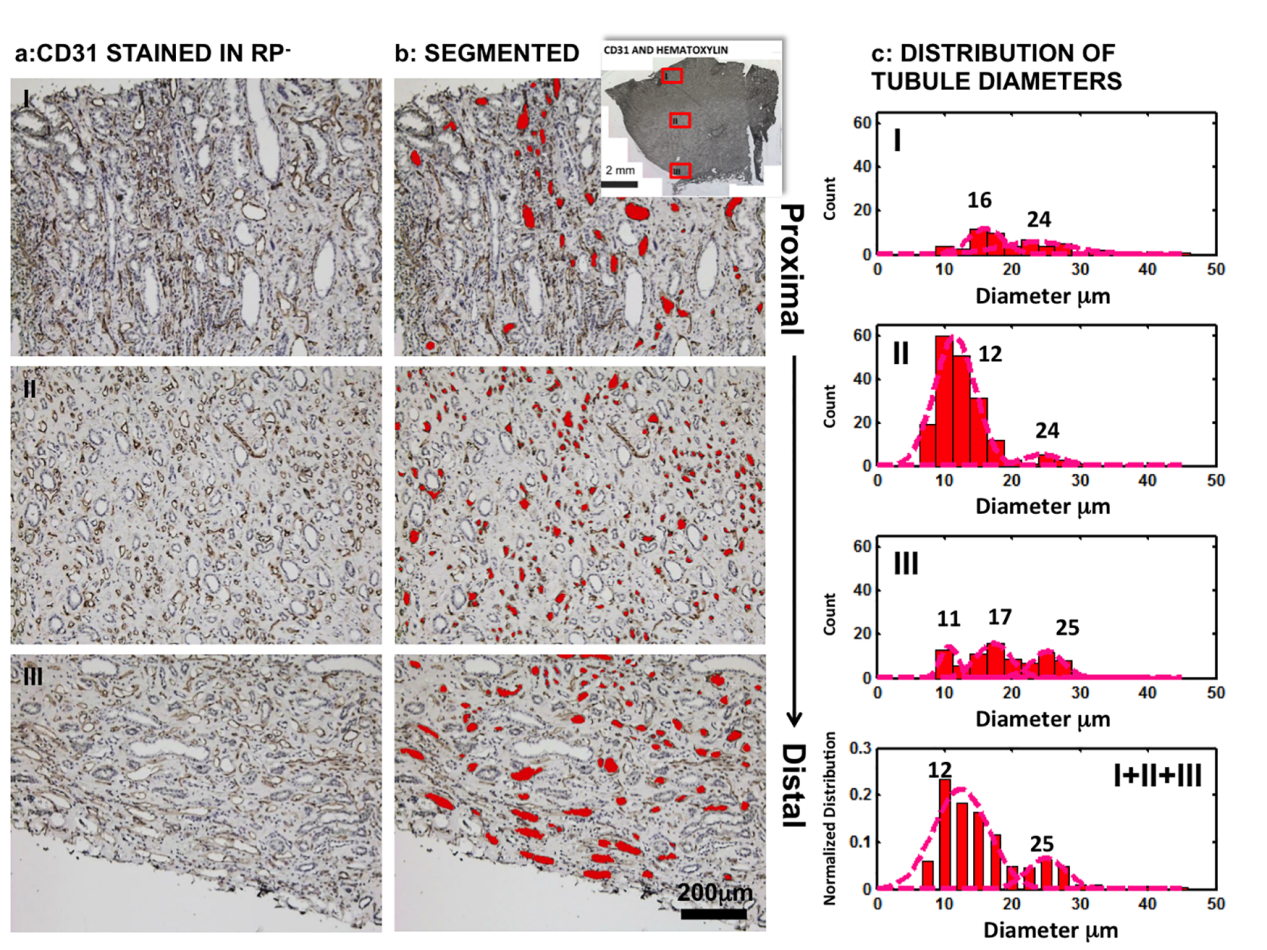
Light microscopy images of histology sections from human papilla without identifiable Randall’s plaque (RP-) immunolocalized for CD31. CD31 was localized around the vasa recta and was digitally segmented (red) to identify diameter range as shown by the zone-specific histograms. The red squares in the inset represent the three zones (I, II, and III) of interest and correspond to the most proximal region of the medullo-papillary complex (I), the mid-region of the complex (II), and papillary tip (III) respectively. The histograms of the CD31 immunostained tubule’s diameter of each zone were plotted separately. The bottom right histogram shows a normalized distribution of stained tubular diameter and was plotted from the summary of all three zones (I+II+III). Among specimens with RP (RP+), many mineralized tubular walls were observed distally in the RP+ specimens (Fig 5). After analyzing the mineralized tubules and vasa recta using the same segmentation approach on histologic sections (Fig 5b), the mineralized tubules and iodine stained tubules illustrated similar diameters. The peak distribution of CD31 immunolocalized tubule diameters in RP+ gave a slightly higher value than that of RP- while the majority of vasa recta from both groups had a diameter between 10–40 μm. Both medullo-papillary and cortico-medullary data is presented as supplemental information (see S3 Fig).

Scanning electron microscopy (SEM) images and energy dispersive X-ray spectroscopy analyses of iodine stained histology sections indicated that the erythrocytes in vasa recta showed significantly higher concentrations of iodine than in other cell types or structures (Fig 3). These observations were evident through point (Fig 3a) and area (Fig 3b) analyses of regions of interest. Presumably the iodine preferentially binds to glycogen in erythrocytes [36, 37], resulting in a higher iodine content of vasculature distinguishable from other neighboring tissue structures. An early report also noticed vasculature especially blood can be intensely stained by iodine [34]. Here the preferential staining of iodine on erythrocytes supports these observations. Further validation was performed by examining a RP- papilla labeled with CD31 (Fig 4a), which highlighted the endothelial layer of vasa recta and allowed digital segmentation for diameter analysis (Fig 4b). These measurements were compared to the diameters of iodine stained tubules from tomograms. The diameters of these CD31 positive tubules typically ranged from about 10–30 μm (Fig 4c) in the three different locations from proximal to distal in the medullo-papillary complex (S1B Fig). Vasa recta labeled with CD31 showed two major distribution peaks between 10 μm and 30 μm (Fig 4c).

**Fig 5:**
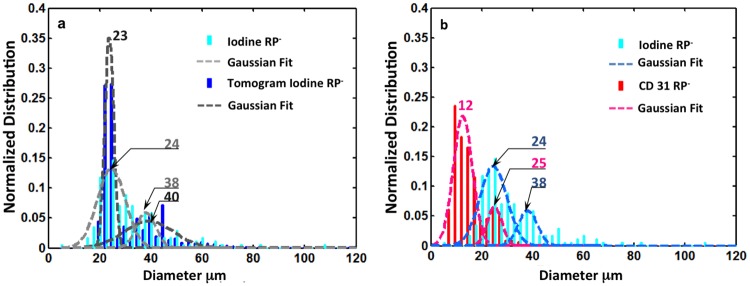
Comparison of tubule diameters visualized using different techniques. (a) Comparison of tubule diameter distribution from iodine stained RP- histology section (cyan) and tomogram (blue). (b) Comparison of the distributions of tubular diameters of RP- histology sections that were stained with iodine (cyan) and immunolocalized for CD31 (red).

### Distal, interstitial minerals in RP+ tissue have additional morphological features that are distinct from proximal intratubular minerals

**Fig 6:**
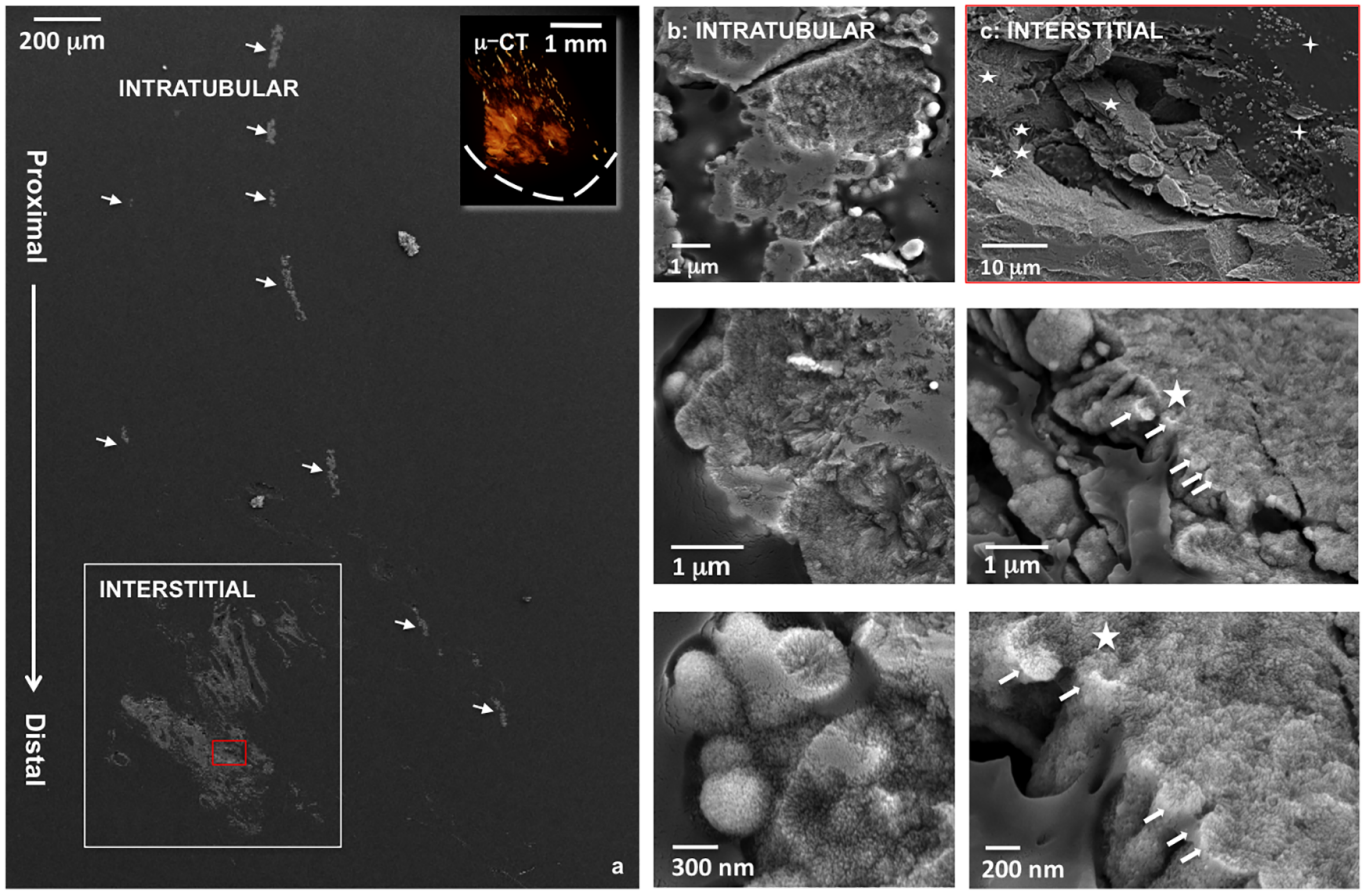
Representative SEM images from the microtomed block surface of a RP+ specimen are shown. Inset on upper right of (a) illustrates 3D digital reconstruction where the dashed line indicates the contour of the papilla. The intratubular minerals are shown in arrows and correspond to images in (b). The interstitial minerals in the lower left inset correspond to images in (c). Despite their distinct locations within the medullo-papillary complex, these regions show subtle differences in structural features. In (b), the proximal intratubular minerals are in the form of spherical aggregates with many dense and thin plate- or needle-like crystals (four-pointed stars). (c) The distal interstitial minerals also showed aggregates with many small crystalline particles embedded in organic matrix (see stars). Some mineralized fibrils (arrows) were revealed at the edge of the aggregates. In addition, many small mineralized nodules (four-pointed stars) were found in the vicinity of large interstitial mineralized aggregates.

Fig 6a illustrates both intratubular and interstitial mineralization on the sectioned surface of a renal medullo-papillary complex (inset in upper right of 6a represents 3D digital reconstruction of the same specimen). Proximal mineralization was intratubular compared to interstitial distal mineralization (Fig 6a). Among the intratubular minerals, many plate-like or needle-like crystals were found inside the tubules (Fig 6b). These intratubular crystals were of several hundred nanometers in length and formed larger spherical aggregates (Fig 6b, from top to bottom). In contrast, spherical nanoparticles (Fig 6c, four-pointed star) coalesced to form larger concretions (Fig 6c, stars) in the collagenous matrix of the interstitium and occupied larger areas of the distal interstitial matrix. These crystals in the intratubular and interstitial region have been identified as apatite and the detailed analysis of the intratubular and interstitial minerals will be reported elsewhere.

### Minerals in the patent tubular walls of RP+ tissues are associated with noncollagenous molecules and exhibit strong autofluorescence

**Fig 7:**
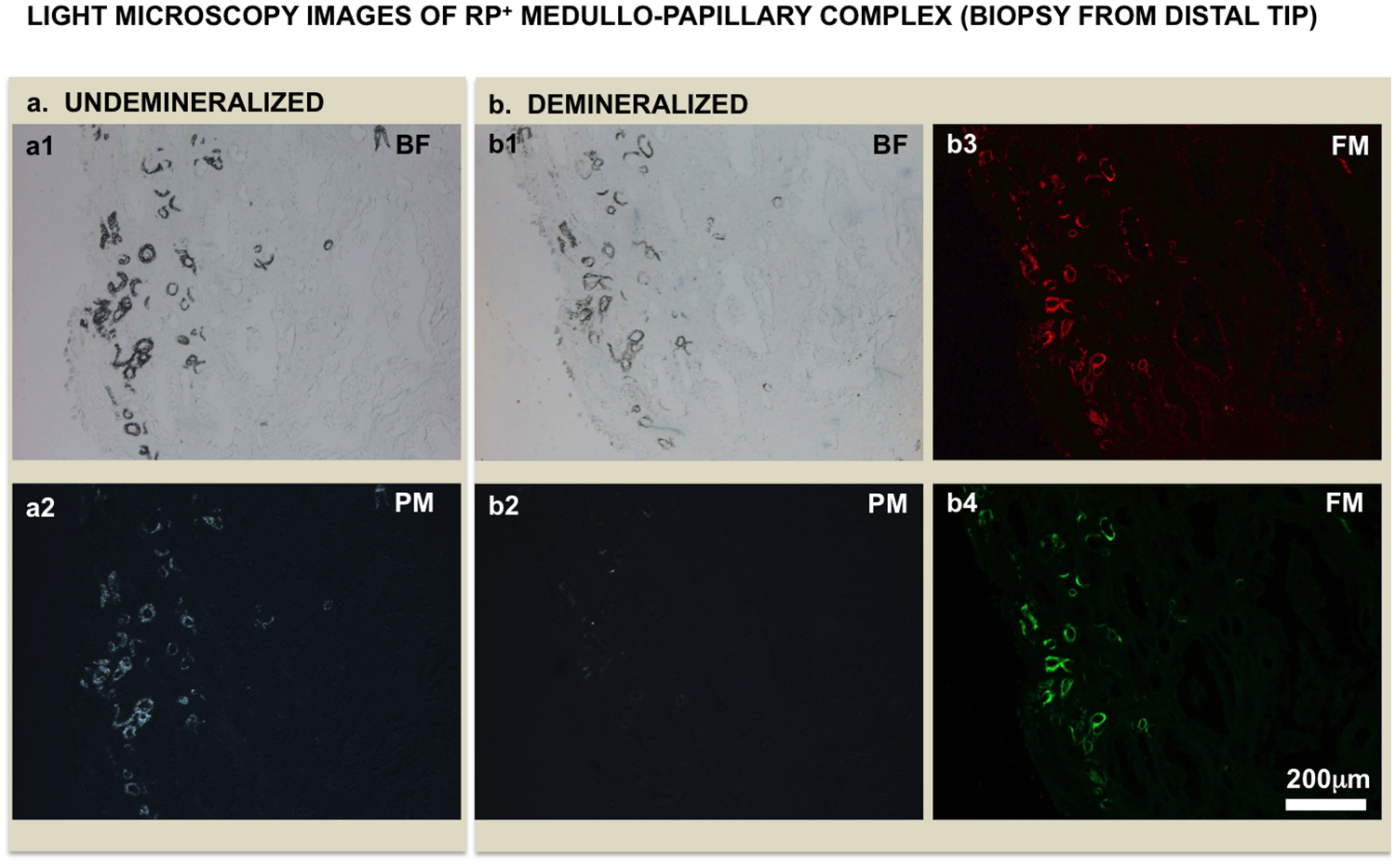
Light microscopy images of RP+ biopsy specimen (a) before and (b) after 4% EDTA treatment for 1 hour. The corresponding polarized microscopy (PM) images were shown in (a2) and (b2) respectively. Birefringence is associated with mineralized tubules. After decalcification using EDTA for 1 hour, weak birefringence is noticeable in some tubules (b2). The mineralized tubules show strong red (b3) and green (b4) autofluorescence using fluorescence microscopy (FM) even after 1 hour EDTA treatment, suggesting that the autofluorescence is associated with organic component while minerals are birefringent. Note that the structure of the lumen changes upon demineralization (compare a1 and b1 micrographs).

Mineralized tubules were clearly observed on histology sections owing to their natural opacity to light (Fig 7a1). After treatment with 4% EDTA for 1 hour, the previously mineralized tubular walls appeared lighter in color (Fig 7b1). The birefringence of these mineralized tubular walls (Fig 7a2) using polarized microscopy images decreased substantially after EDTA treatment (Fig 7b2). However, after the demineralization process, there was retention of strong red and green autofluorescence in mineralized tubules suggesting that birefringence could be associated with minerals while the organic noncollagenous macromolecules could be responsible for autofluorescence (Fig 7b3 and 7b4).

### The walls of patent tubules in RP+ papillae were immunopositive for several noncollagenous proteins

**Fig 8:**
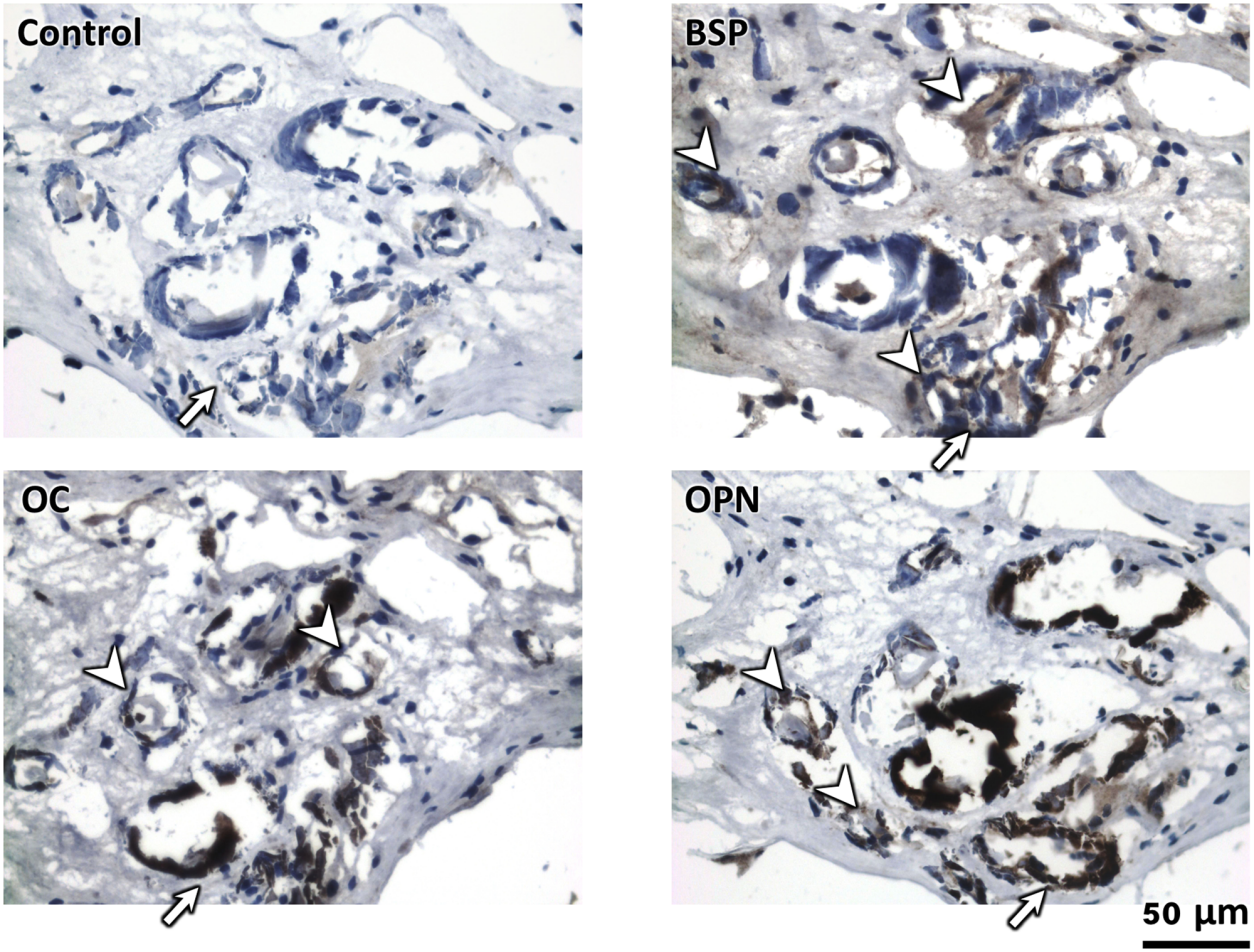
Light microscopy images illustrate immunolocalized regions in RP+ specimen. Light micrographs provide representative immunolocalized regions for three non-collagenous proteins (BSP, OC, OPN) and a control. These non-collagenous proteins appear to be associated with mineralized tubules (arrows) and vasa recta (arrowheads). BSP: bone sialoprotein; OC: osteocalcin; OPN: osteopontin.

**Fig 9:**
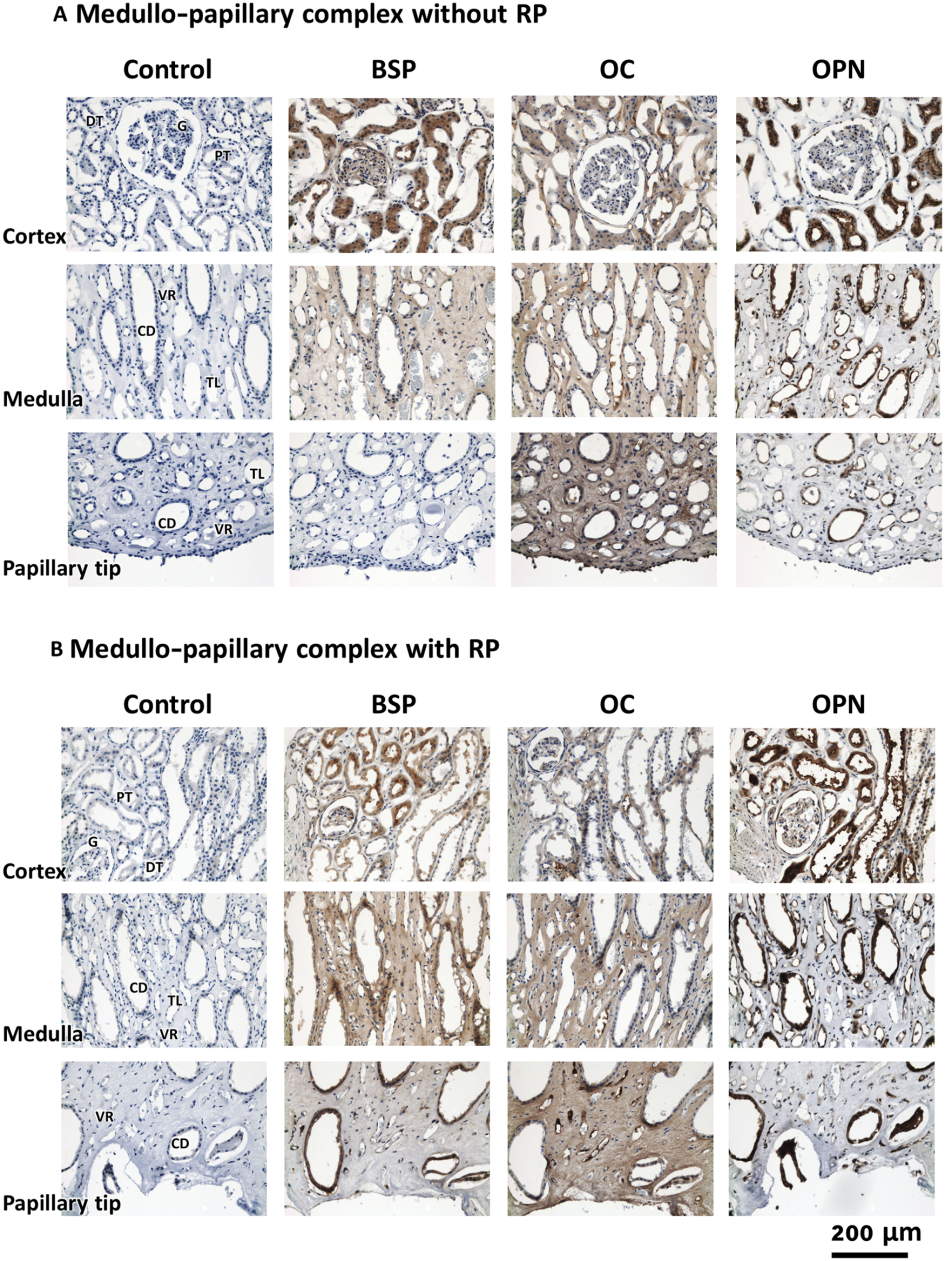
Light microscopy images illustrate regions immunolocalized for noncollagenous proteins (NCPs) of a medullo-papillary complex a) without and b) with Randall’s plaque. The representative three sections from cortex to distal tip were immunolocalized. The control with no primary antibody is also included. All NCPs show positive staining in proximal cortex regions. Irrespective of the presence or absence of RP in the renal papilla, BSP, OPN and OC can be identified in the renal tubules and vasa recta in the functional zones of the renal papilla. However, no BSP is labeled in the distal region of RP- papilla. Both BSP and OC are labeled in the interstitium where the staining of OPN is weak or negative, especially in the regions of medulla and distal tip. G: glomerulus; DT: distal tubule, PT: proximal tubule; CD: collecting duct; TL: thin limb; VR: vasa recta.

**Fig 10:**
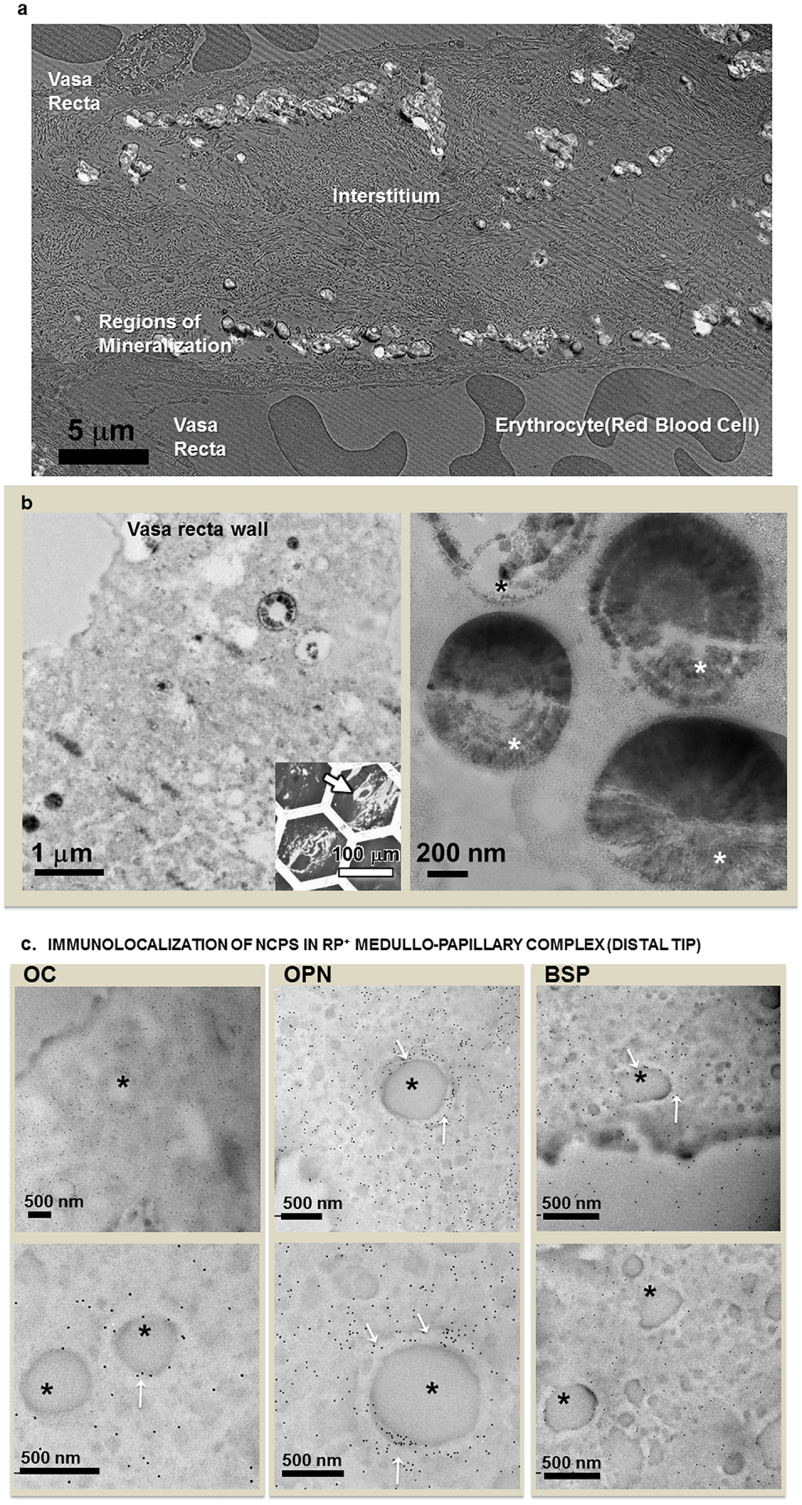
TEM micrographs at various length scales are shown to highlight the specificity of NCPs. (a) Image of mineralized nodules in the regions associated with vasa recta at the papillary tip of a RP+ tissue. These nodules appear to locate within the basement membrane of endothelium and adjacent regions. (b) TEM images of mineralized tubular walls of tens of microns illustrate crystalline aggregates and spherical nanoparticles (asterisks) at the papillary tip of a RP+ tissue. No cellular structures were identified in these mineralized tubular walls. That is, morphological features of the endothelium/epithelium are not visible. The spherical nanoparticles show a layered structure. Inset on bottom right provides a light micrograph indicating the location of a representative mineralized tubule (arrow) from which TEM images were taken. (c). TEM images illustrate immunogold labeled thin sections of mineralized tubular wall of RP+ tissue. The profile of nanoparticles’ spherical shape is discernible under TEM due to intense labelling for OPN and OC in the mineralized vasa recta wall and the surface (arrows) of nanoparticles (asterisks). Lower panel images were taken at higher magnification in a different location.

Light microscopy images of demineralized RP+ sections immunostained with NCPs such as OPN, OC, and BSP suggest their strong localization in the mineralized walls of patent tubules (Fig 8). BSP and OC were contained more in the matrix than OPN. The intensity of labeling of BSP appeared higher in the mineralized vasa recta wall (arrow heads) than in the interstitial regions. The walls of the vasa recta also showed higher staining than tissue background. On RP- and RP+ tissue sections, strong OPN expression was found in vasa recta encircling renal tubules within the proximal medullo-papillary complex (Fig 9). The interstitium of distal region shows more BSP on RP+ (Fig 9b) than RP- (Fig 9a). OC was also found in interstitium as well as renal tubules and vasa recta. Some of the tubular cells also showed positive staining of NCPs. Table 1 summarizes the distribution of the NCPs in different anatomical regions of medullo-papillary complex with and without RP. Ultrastructural examination of RP+ using a TEM revealed areas of layered spherical nanoparticles in the crystalline aggregates of mineralized tubular wall associated with vasa recta (Fig 10a and 10b). The morphological features of the spherical nanoparticles were similar to that previously reported by others [7, 38]. TEM images of immunogold labeling of OC, OPN, and BSP on the demineralized RP+ tissue sections showed intense labeling in the walls of the patent tubules (Fig 10b). The profile of the nanoparticles was preserved after demineralization and many gold particles were found on the surface or the outmost layers of the nanoparticles (Fig 10c). These observations of OPN and OC immunostaining were similar to earlier publications on RP or kidney stones [16, 27, 28]. Although more intense labeling of BSP was found in the mineralized tubular wall, many gold nanoparticles were also found in the lumen and interstitial region adjacent to tubules at the tip of the renal papilla. The results from light microscopy, correlative microscopy (using fluorescence and electron microscopy), and TEM are complementary, indicating the precision of the site-specific immunolocalization of proteins.

**Table 1:** Immunolocalization of NCPs in different anatomical regions of medullo-papillary complex with and without RP.

NCPs	Medullo-papillary complex without RP			Medullo-papillary complex with RP		
	Cortex	Medulla	Papillary tip	Cortex	Medulla	Papillary tip
BSP	G,PT	CD,TL,VR,IS	-	G,PT,DT	CD,TL,VR,IS	CD,TL,VR,IS
OC	G,PT	CD,TL,VR,IS	CD,TL,VR,IS	G,PT,DT	CD,TL,VR,IS	CD,TL,VR,IS
OPN	G,PT	CD,TL,VR	CD,TL,VR	G,PT,DT	CD,TL,VR,	CD,TL,VR

G, glomerulus; PT, proximal tubule; DT, distal tubule; TL, thin limb of loop of Henle; CD, collecting duct; VR, vasa recta; IS, interstitium.

## Discussion

Mineralized papillary tissues in the kidney clinically identified as RP, are believed to be the nidus from which most calcium-based kidney stones form [7, 8]. In this study, results: 1) illustrated detectable amounts of mineralization within tubules more proximally within the medullo-papillary complex, away from the commonly investigated papillary tip; 2) indicated a plausible involvement of vasa recta in mineralization of the complex; 3) provided insights into the role of NCPs towards pathologic mineral formations; and 4) through correlative and complementary microscopy at multiple lengths scales, contextual information of the nanoparticles was established.

While there have been no known prior studies evaluating the proximal intratubular mineralization within the medullo-papillary complex, the cause for interstitial mineralization has several postulates. These include the oxidative stress theory [8] and differentiation of renal cells to osteoblast-like phenotypic [12]. The latter hypothesis may stem from the evidence that several NCPs have been found in RP [27, 28] and kidney stones [16] albeit their environments being significantly different. In bone, these NCPs are closely correlated with osteoblast, osteoclastic activities in maintaining the quality needed for bearing loads. Another hypothesis involves interstitial mineralization through accumulation of NCPs at the papillary tip by way of the vasa recta [39]. Other factors, such as supersaturation, change of pH, and matrix vesicles with lipid, may play an important role regulating the mineralization in renal papilla [18–20].

### Intratubular and interstitial mineralizations are also associated with vasculature in the renal papillae

Histomorphometry through post analyses of 3D digital reconstructions of whole human medullo-papillary complexes from micro-CT (S2 Fig) indicate prevalence of proximal mineralization in both RP- and RP+ tissues (Fig 1 and S3 Fig). By identifying the anatomical location and evaluating the size of mineralizing tubules, iodine stained tubules, and CD31 positive vasa recta, it is conceivable that these proximal intratubular minerals are located within vasa recta for both RP- and RP+ tissues. Further analyses of the histology sections from RP+ tissue, specifically those immunolocalized for CD31 whose diameter was compared to iodine positive tubules (Fig 5) confirmed that the mineralized tubular walls at the papillary tip are primarily mineralized vasa recta. The intratubular mineralization of vasa recta in the proximal medullo-papillary complex discovered in this study has not been reported previously, and its consistent finding across all specimens is significant. These results support the theory of the involvement of vascular elements towards kidney stone formation [39]. It should be noticed that results from this study do not contradict, but strengthen the argument in some of the earlier findings through evidence that the mineralization can occur in both urinary tubular epithelium and vasa recta (Figs 1–6 and S3 Fig). The histology sections of RP+ medullo-papillary complexes show mineralized vasa recta where endothelial cells cannot be observed. From light and electron microscopy techniques, and micro-CT data (all figures), it is plausible that mineralization could have occurred in some tubules larger than vasa recta, and within the interstitial tissue between these tubules.

It is interesting to note that proximal mineralization is limited to the tubules, while distal mineralization is within the interstitium leaving the tubules patent (Figs 1 and 6 and S3 Fig). Regardless, the other common denominator is the role of the same NCPs and their plausible implication as nidi for biomineralization to occur inside a tubule, and outside in the interstitium (extracellular matrix) in distinct anatomical locations of the renal papilla. It is likely that intratubular and interstitial mineralizations could be due to different cascades of events, albeit morphologically and chemically similar nanoparticles could be the building blocks. Various charged proteins in blood are responsible for inhibiting mineralization and maintaining homeostasis by binding to calcium and other ions. These proteins regulate the ion concentration in blood; however, pathologic vascular mineralization can occur if the balance between free and bound ions is interrupted. The supersaturated condition in blood, although being at lower quantities in the distal part of the renal papilla (triggered by disruption of metabolic processes as fundamental as dehydration followed by hydration), could facilitate nucleation and growth of the observed apatite crystals [40]. Previous reports on intratubular mineralization focus on loop of Henle and collecting ducts. Apparently, the continuous exchange of ions, water, and other macromolecules between urine and blood through epithelium, interstitium and endothelium can cause the supersaturation in various locations. Therefore, mineralization is not necessarily limited to the epithelial basement membrane [18–20]. Compared to urine, the higher pH in blood favors formation of apatite minerals.

### Noncollagenous proteins are not anatomically-specific

Previous studies have found that mineralization occurs in the basal membranes of uriniferous tubules and vasa recta at the papillary tip [38]. NCPs such as OPN have been associated with the mineralized plaques of RP [27, 28]. These results indicate that NCPs can accumulate in the basal membranes during the fluid exchange between renal tubules and neighboring vasa recta. The locations of NCPs are not associated with a specific cell type or tissue structure. Therefore, delivery of NCPs are postulated to be through vasculature though these NCPs could be synthesized by cells including those located within the uriniferous tubules, or fibroblasts of the interstitium, or even other sources. Adhesive and cohesive interactions between charged NCPs and ions can occur, which in turn facilitate nucleation and subsequent growth into the observed nanoparticles often thought to be the precursors for stone formation. Therefore, the structures featuring higher concentration of NCPs also represent the accumulation of calcium ions. It can be expected that these regions tend to mineralize earlier than neighboring sites. In fact, vasa recta and epithelial cells of some the uriniferous tubules in RP+ specimen show either positive or stronger localization of NCPs than that observed in the background within the same tissue section (Fig 9) and corroborates with earlier reports [16, 28]. Meanwhile, the positive staining of these tubular cells may indicate an active intracellular synthesis of NCPs, which is in accordance with the hypothesis of osteoblast phenotype change in the distal region [12, 41]. However, the strong expression of BSP, OC, and OPN in vasa recta and other tubules (Fig 9) belonging to the proximal medullo-papillary complex suggests a different source of NCPs, that is, NCPs may be delivered from proximal renal tubules or vasa recta to the distal region. OC has been reported to circulate in blood and have many other physiologic functions [42–45] in addition to mediating mineralization [16, 25, 26, 46–48]. OPN is also associated with inflammatory diseases and has been identified in blood [49]. Upregulated synthesis of OPN in epithelial cells and macrophages were associated with quite a few renal diseases [50], indicating the multifunction of OPN. Serum BSP has been proposed to serve as a biomarker associated with several bone diseases [51, 52] and it has not been identified in mineralized papillary tissues from *in vivo* studies [16]. It remains a question whether the uriniferous cells simply help transport some NCPs during fluid and ion exchange with vasa recta via endocytosis-exocytosis pathway or undergo a phenotypic change and synthesize all these NCPs especially in the distal region. Regardless, both scenarios can give positive immunostaining of NCP within tubular epithelial cells. Interestingly, the thickness of the mineralized wall is similar to the thickness of an epithelial lining that is commonly observed in histology sections. This implies that lipids from the degraded cells can also act as nucleators giving the “onion-ring” like appeal to the observed nanoparticles [53]. However, within the observed histology sections, the spaces between the rings were positive for the NCPs (Fig 10).

### Noncollagenous proteins mediate mineralization

Localization of NCPs in mineralized tubules of RP+ tissue suggests a strong correlation between NCPs and the mineralized nanoparticles. NCPs are ubiquitous in organic tissues and normally aid in favorable biomineralization [54–60]. The question that arises when investigating proteins is not only their presence, but if the detected protein has a functional motif, that would then allow aggregation of inorganic ions. In general, mammalian BSPs are phosphoproteins with an open flexible structure in which two polyglutamic acid domains interact with apatite [61]. BSP has many functions including nucleating apatite crystals [62] and regulating osteoclast differentiation and activities [63, 64]. It should be noted that BSP has not been identified in RP nor calcium based kidney stones from *in vivo* studies [16]. To date, the upregulated expression of BSP has only been observed from an *in vitro* system in which renal cells were cultured in a media rich in calcium. The structure of OPN is rich in serine and glutamic acid with a characteristic polyaspartic motif [65]. The acidic serine- and aspartic acid-rich motif (ASARM) has been hypothesized to regulate mineralization [64, 66–68]. Although these charged macromolecules appear to inhibit the calcification in general, the high affinity of calcium ion to charged organic macromolecules may suggest different roles including inhibition and facilitation of heterogeneous nucleation and growth of calcium based crystals.

Based on principles of thermodynamics, a recent study also suggested that the flexible structure of intrinsically disordered NCPs is crucial for the mineralization [69]. An *in vitro* study suggested that the simultaneous synthesis of BSP and OPN by renal epithelial cells in the inner medullary collecting duct can result in calcified nodules. Several groups have reported the localization of OPN either in RP+ tissues or renal calculi [16, 28]. However, the localization of BSP towards intratubular and interstitial mineral formations within the renal papilla has not been reported. OC, another abundant bone matrix protein, has been localized in renal calculi but no publications have shown its anatomical specificity and appearance in RP.

With OC, the γ-carboxyglutamic acid groups are responsible for binding of calcium ions as well as to apatite crystals [25, 70, 71], and is thought to regulate collagen mineralization in calcified connective tissues such as bone and tendon [35, 72]. The results from this study suggest that pathologic intratubular and interstitial biomineralization in the medullo-papillary complex could be facilitated by NCPs, although their origin and mechanistic process is yet to be investigated. Given their ubiquitous existence in normal mineralized tissues, whether these NCPs promote or inhibit the pathological mineralization is still unclear. Early *in vitro* studies of individual NCPs suggest OPN and OC can inhibit or delay apatite crystal formation, while BSP facilitates nucleation [73]. However, the strong immunostaining of BSP in the distal region of RP+ tissue in both light and electron microscopy images suggests the role of BSP in tubular mineralization.

The exact roles of NCPs are elusive due to naturally built in functional redundancy in NCPs and their compensatory behavior in the absence of another [74–76]. NCPs usually show multiple functions that are determined by their structure and isoforms after post-translational modifications. However, it remains to be a challenge to localize the exact isoforms. Transgenic technology using *in vivo* models [63, 77, 78] has been exploited to investigate the effect of an individual NCP, in addition to *in vitro* studies [62, 73]. The renal mineralization has also been addressed in a few studies using knockout mouse models. It was reported that OPN favors formation of calcium oxalate dihydrate while many intratubular calcium oxalate monohydrate crystals are found in OPN-deficient mice [79]. Similar intratubular minerals were also noticed in Tamm-Horsfall protein deficient mice [80]. However, for mice deficient of other NCPs such as BSP and OC, their effect on pathological mineralization in renal papilla is yet to be documented. Although genetically modified animals would give a better clue for the role of these proteins in biomineralization of renal tissue, it should be noted that nature is conserved and that there also appear to be several compensatory processes by which biomineralization can still be facilitated in tissues. As such, tissue specific transgenic could be a viable technology to investigate the role of NCPs, but in conjunction with MMPs and TIMPS as dystrophic mineralization results from an orchestrated effort between various key primary molecular events [81]. As for OPN and OC, their negatively charged groups such as aspartic acid and glutamic acid bind to the positively charged surface ions such as calcium of apatite crystals and inhibit crystal growth by preventing addition of other negative ions such as phosphate. The results of immunogold labeling suggest that the further growth of nanostones may be inhibited by the two NCPs. Nevertheless, *in vitro* studies suggest that OPN and OC can form a complex and promote mineral formation [82, 83]. Therefore the co-localization of OPN and OC together with BSP may be one of the causes for the tubular mineralization in RP+ tissue. Similar observations were also found in kidney stones where OPN and OC co-localize within the internal layers of the stone [16]. The structural and compositional similarities between kidney stones and nanostones found in RP+ specimen suggest an overlap in their mineralization mechanisms. The identified NCPs allude to a plausible change in phenotype of the proximal medullo-papillary tissue toward bone-like phenotypes, also observed in mineralizing zones in other organ systems such as the breast, teeth, and bone, and subsequently in larger forms can cause impairment of function.

## Conclusions

The histomorphometric analyses at multiple length scales has allowed us to associate mineralization within the two types of tubules, however, the biomineralization process is yet to be investigated. Results from this study indicated that vasa recta may also be affected by vasculature mineralization which is dominant in the interstitial region, while intratubular mineralization in the proximal regions of the medullo-papillary complex of the renal papillae could be vascular and/or urothelial tubules—the functional units of the renal papilla. Future studies will link intratubular with interstitial mineralization within the medullo-papillary complex using the tenet, form guides function [84].

## Supporting information

S1 File Supporting method information for tubule analysis. (DOCX)

S2 File Supplemental video shows iodine stained blood vessel (blue) in line with tubular mineralizations (green). (MPG)

S1 Fig Montage of high resolution light micrographs of histology sections stained with (a) iodine and hematoxylin, and (b) CD31 and hematoxylin are shown. The red squares represent the three zones (I, II, and III) of interest and correspond to the most proximal, the mid-region of medulla, and the tip of the medullo-papillary complex.
(TIF)

S2 Fig Diameter distribution of various tubules in 5 regions along medullo-papillary axis based on tomogram of a RP- papilla. (a) Mineralized tubules (green) in unstained specimen, (b) iodine stained tubules (blue), (c) void tubules (grey) in the stained specimen following digital inversion, (d) stained (blue) and void (grey) tubules are shown. The tubules with larger diameters are highlighted. The red blob is an imaginary kidney stone at the papillary tip.
(TIF)

S3 Fig Flow chart for calculating tubule diameter. I: The void area in a 3D volume is marked as a tubule. II: Segmentation of tubules. III: The 3D volume is rotated to a direction which is most perpendicular to all the tubules. IV: Slices are generated one by one along this direction. V: The tubule diameter (D1, D2 and D3) is determined by the minor axis length of the smallest eclipse which can cover all the pixels of the tubular cross section as show by the blue lines.
(TIF)
