# Supplementary material for: Anatomically-specific intratubular and interstitial biominerals in the human renal medullo-papillary complex
Source: PLoS One. 2017 Nov 16;12(11):e0187103. doi: 10.1371/journal.pone.0187103 (PMC5690653; doi:10.1371/journal.pone.0187103)
Supplement: S1 File — (DOCX) [file pone.0187103.s001.docx]

1. **Tubule diameter analyses** were performed using custom built MATLAB (R2013b, MathWorks. Natick, MA) code. Conceptually, the algorithm involves extracting diameters of tubules by processing data gathered from two-dimensional slices (2D) using light microscopy and 2D-virtual sections from X-ray tomograms. Conventional and virtual histology sections were used to extract tubule diameter.

To evaluate tubule diameter, 2D virtual sections of the 3D tomograms obtained from micro-CT were binarized using AVIZO, where pixels designated as ‘1’ represented the area of interest (either minerals or tubules), and pixels designated as ‘0’ represented other interstitial materials (Fig.S2A-I, II). The 3D matrix extracted from the tomogram was spatially transformed to be most parallel to the direction of stained or mineralized tubules (Fig.S2A-III). Slices perpendicular to this direction were generated one by one to calculate the diameter of tubules/mineral using the minor axis length of the smallest ellipse which could cover all the pixels of a tubule section (Fig.S2A-IV, V). This approach was applied to all slices within the 3D tomogram to generate a normalized distribution plot of diameters of tubules. The number of tubules of different diameters was plotted as a histogram. Normalized distributions of mineralized and iodinated tubules were obtained by first dividing the number of tubules at a specific calculated diameter range with the total number over the entire diameter range. The relative ratios of the tubules at each diameter range were plotted. Subsequently, the normalized diameters of mineralized tubules, vasa recta immunolocalized for CD31, and iodine stained tubules were analyzed and compared. Tubular diameter analyses were performed using customized MATLAB code.

Fundamentally, light micrographs are in a RGB format, i.e., each figure is a superposition of light intensities with three representative 2-D matrices of ranges from 0 to 255 that can be related to the brightness of red, green, and blue emitting from a micrograph respectively. Since the stained tubules are either yellow or brown (lower intensities of blue), we used blue matrix to segment the iodine-stained tubules by selecting the darker pixels using AVIZO (9.0.1, FEI, Hillsboro, Oregon). Following segmentation, a binary 2D matrix (containing 0s and 1s) was obtained. In this matrix, pixels numbered “1” represent tubules while pixels numbered “0” represented other parts of the tissue such as interstitium. Then tubule diameters were extracted from the 2D matrix using the same “eclipse cover” method described previously. The normalized distribution of the extracted diameters was also plotted.

1. **Distribution of tubule diameters in the medullo-papillary complex:** In addition, diameter analysis of RP^-^ papillae along the medullo-papillary axis based on tomography was also plotted in Fig. S3. For unstained specimens, mineralized tubules in general showed much larger diameters located at the tip of papillae, though some larger intratubular minerals were observed near the cortex (Fig.S3A). In the stained specimen, the diameter distribution was almost identical at all ranges (0-40µm) along the medullo-papillary direction (Fig. S3B). More tubules with larger diameters were at the cortex and were predominantly comprised of intratubular mineral instead of the iodine stained tubules. After taking an inverse of the X-ray tomogram (same data set), the void tubule diameter distribution showed a similar appearance, except that sections near the cortical region had an additional major distribution of diameters at 140~250µm range (Fig. S3C). These imaginary tubules filling the original voids were consistent with iodine stained tubules (Fig. S3D), suggesting that the void tubules and the stained tubules are both vascular in origin. That is, these larger tubules are renal arteries/veins (see Fig. 10) in the proximal portion of the medullo-papillary complex from which the iodine stain (red blood cells) was washed away during sample preparation. In summary, the diameters of tubules with minerals either inside tubules or those extracted from inverse-tomogram are within the same diameter range as the diameters of vasa rectae preferentially stained with iodine.
