# Supplementary figures and images for: Anatomically-specific intratubular and interstitial biominerals in the human renal medullo-papillary complex
Source: PLoS One. 2017 Nov 16;12(11):e0187103. doi: 10.1371/journal.pone.0187103 (PMC5690653; doi:10.1371/journal.pone.0187103)

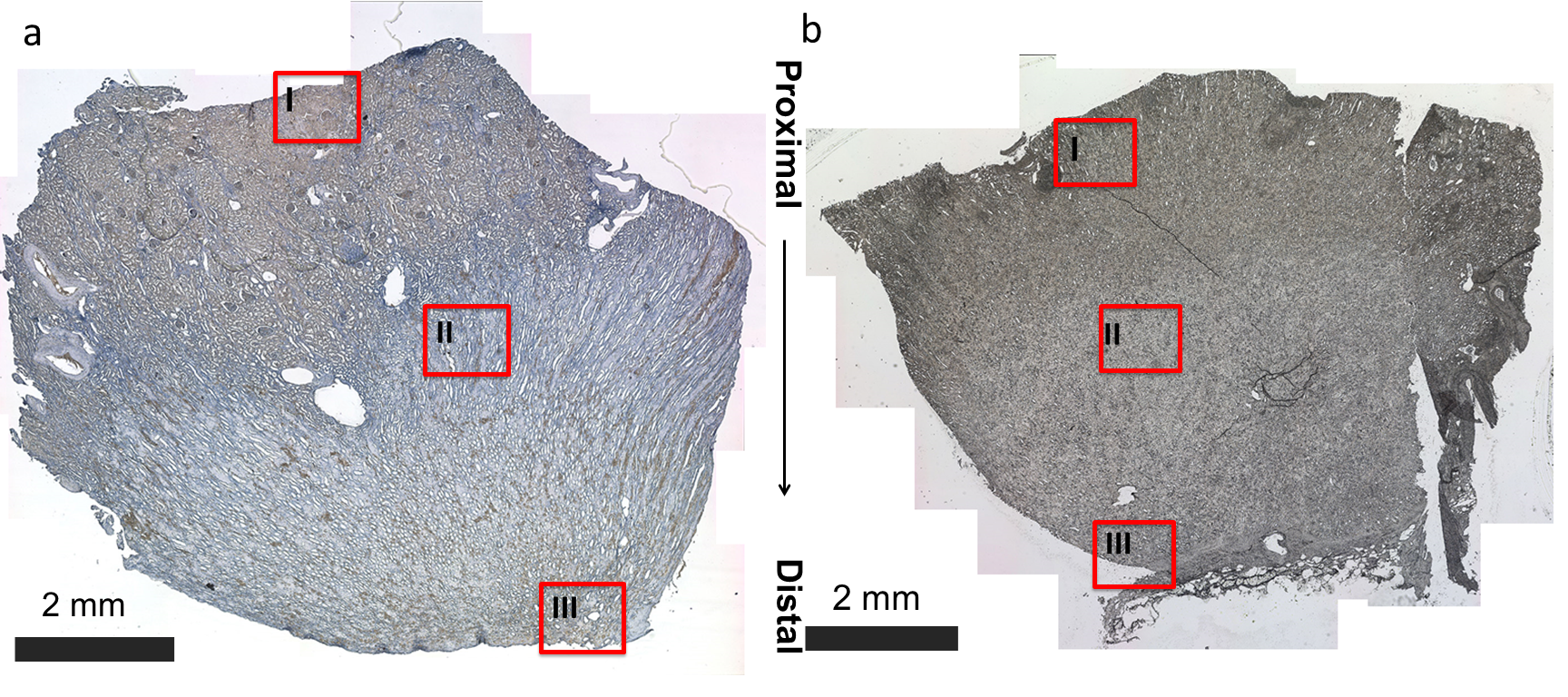

Supplement: S1 Fig — The red squares represent the three zones (I, II, and III) of interest and correspond to the most proximal, the mid-region of medulla, and the tip of the medullo-papillary complex. (TIF) [file pone.0187103.s003.tif]

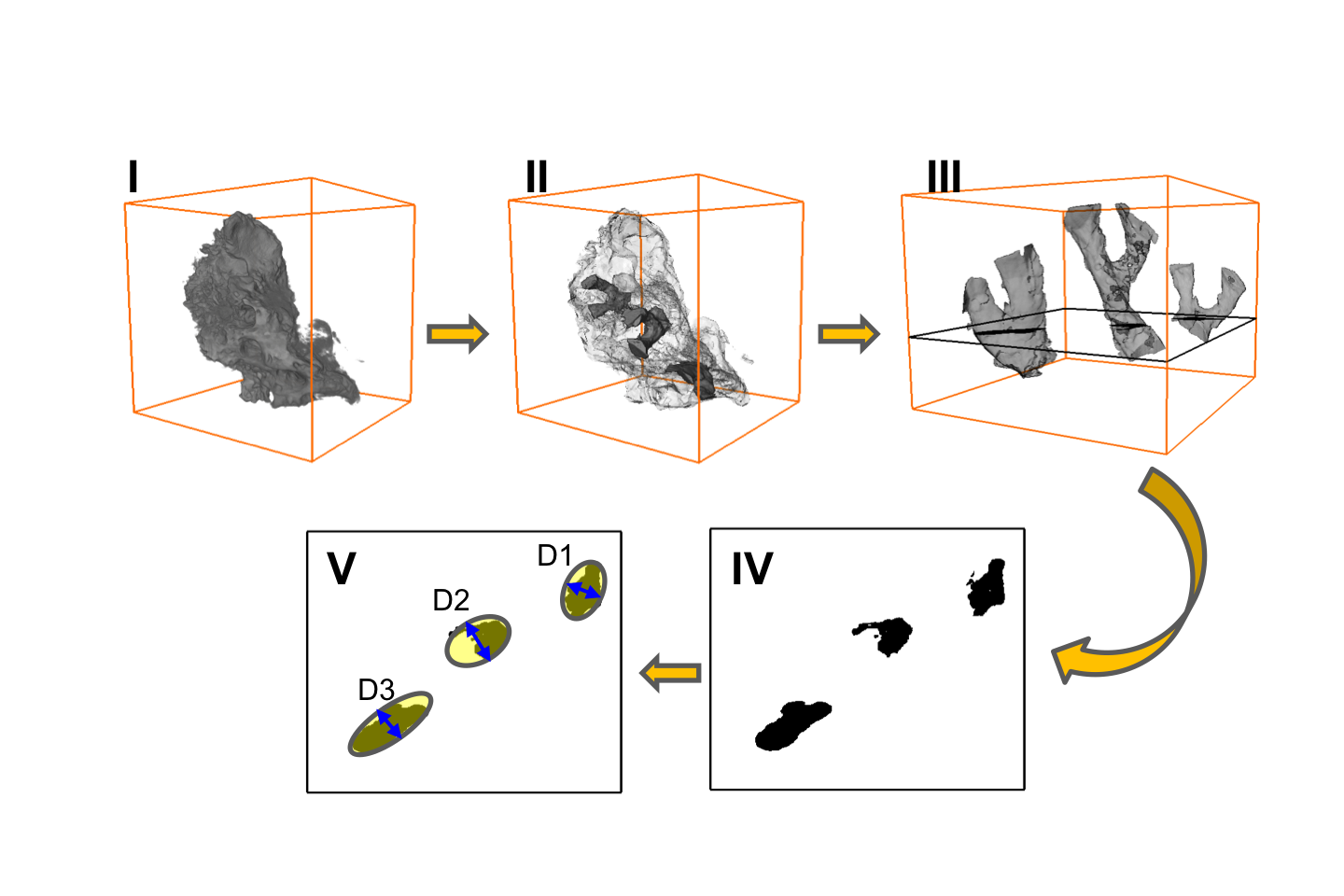

Supplement: S2 Fig — (a) Mineralized tubules (green) in unstained specimen, (b) iodine stained tubules (blue), (c) void tubules (grey) in the stained specimen following digital inversion, (d) stained (blue) and void (grey) tubules are shown. The tubules with larger diameters are highlighted. The red blob is an imaginary kidney stone at the papillary tip. (TIF) [file pone.0187103.s004.tif]

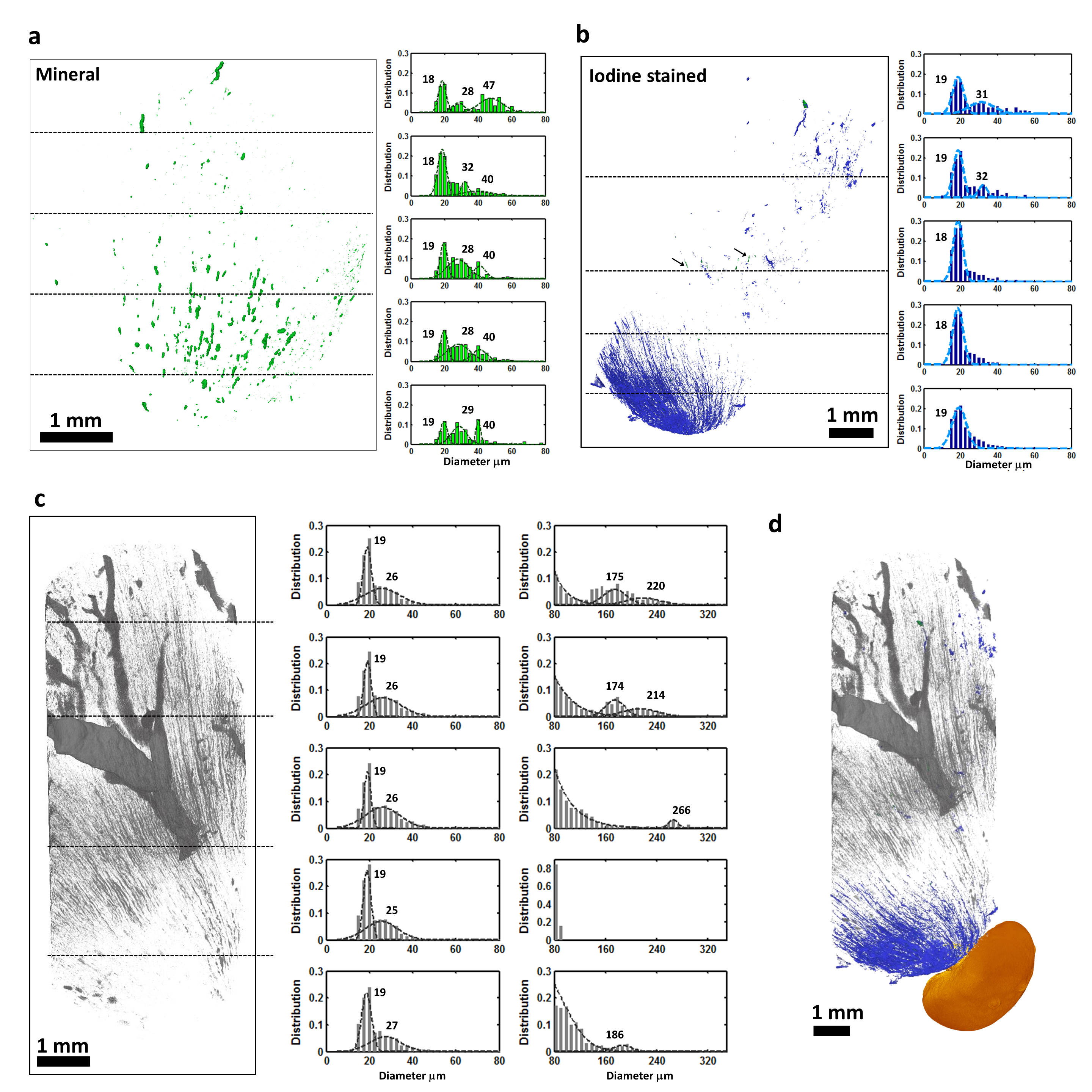

Supplement: S3 Fig — I: The void area in a 3D volume is marked as a tubule. II: Segmentation of tubules. III: The 3D volume is rotated to a direction which is most perpendicular to all the tubules. IV: Slices are generated one by one along this direction. V: The tubule diameter (D1, D2 and D3) is determined by the minor axis length of the smallest eclipse which can cover all the pixels of the tubular cross section as show by the blue lines. (TIF) [file pone.0187103.s005.tif]
